# Who should take care of me? Preferences of old age individuals for characteristics of professional long-term caregivers: an observational cross-sectional study

**DOI:** 10.1186/s13104-017-2717-3

**Published:** 2017-08-10

**Authors:** André Hajek, Thomas Lehnert, Annemarie Wegener, Steffi G. Riedel-Heller, Hans-Helmut König

**Affiliations:** 1 Department of Health Economics and Health Services Research, Hamburg Center for Health Economics, University Medical Center Hamburg-Eppendorf, Hamburg, Germany; 2 Institute of Social Medicine, Occupational Health and Public Health, University of Leipzig, Leipzig, Germany

**Keywords:** Need for care, Preferences, Long-term care, Old age, Nursing home care, Germany

## Abstract

**Background:**

It is most likely that the need for long-term care increases considerably in the next decades due to demographic shifts. Thus, we aimed at identifying the preferences for characteristics of professional long-term caregivers among old age individuals in Germany.

**Methods:**

Data were gathered from a population-based survey of the German population aged 65 and above in 2015 (n = 1006).

**Results:**

It was important for individuals in old age that long-term caregivers were ‘empathetic, kind’ (99.3%), ‘punctual, reliable’ (98.2%), have an ‘orderly appearance’ (96.4%), work in a ‘small team’ (92.5%) and have ‘enough time’ (91.5%). Moreover, while most of the individuals (76.5%) reported high preferences for ‘German language’, the preferences were lower for ‘same cultural background’ (54.2%) and ‘same gender’ (35.7%). In multiple logistic regressions, preferences for ‘same gender’ were positively associated with being female [OR 8.3 (5.6–12.1)], living with partner or spouse [OR 1.4 (1.0–1.9)], and being born abroad [OR 1.8 (1.1–3.1)]. Preferences for ‘German language’ were positively associated with being female [OR: 1.5 (1.1–2.1)]. Preferences for ‘same cultural background’ were positively associated with age [OR 1.0 (1.0–1.1)], living with partner or spouse [OR 1.4 (1.0–1.9)], and East Germany [OR 1.9 (1.3–2.7)]. Preferences for ‘orderly appearance’, ‘empathetic, kind’, ‘punctual, reliable’ and ‘small nursing team’ were all not significantly associated with included independent variables, whereas preferences for ‘enough time’ were positively associated with being female [OR 1.9 (1.1–3.5)], living with partner or spouse [OR 1.9 (1.1–3.4)], education [Apprenticeship, full-time vocational school, OR 3.1 (1.3–7.6)], not providing care for family/friends [OR 1.9 (1.1–3.3)], and involvement in the issue of need for care [OR 1.3 (1.1–1.6)].

**Conclusions:**

Our data suggest that it is important to almost every individual aged 65 and above in Germany that professional long-term caregivers are (i) empathetic, kind, and understanding, (ii) punctual and reliable, (iii) have enough time (e.g., for conversation), (iv) and have an orderly appearance. Furthermore, several factors such as gender or region were found to be associated with preferences for characteristics of caregivers. By knowing these factors, nursing services can tailor their activities to the needs of care-recipients.

**Electronic supplementary material:**

The online version of this article (doi:10.1186/s13104-017-2717-3) contains supplementary material, which is available to authorized users.

## Background

It is most likely that the number as well as the proportion of old age individuals will considerably increase in the upcoming decades [1]. Since age is strongly associated with the need for care [2, 3], it is also most likely that the number of individuals in need for care will markedly increase, posing great challenges.

While it is well known that individuals prefer to be cared for by family members at home as long as possible [4]—which is often explained by factors such as social relations or familiar environments—preferences shift towards professional care and nursing home facilities when the need for care grows [5–8]. However, only a few studies have examined preferences for characteristics of professional long-term caregivers [9–11].

Hence, we aimed at examining which factors are associated with preferences for characteristics of professional long-term caregivers among individuals in old age (65 years and above). Previous studies found that, besides professional caring skills, soft skills such as empathy or sensitivity are important caregiver characteristics for care-recipients [9–11]. With increasing care needs, the specific tasks become more complex and time-consuming [12]. Consequently, when care needs grows, professional caring skills might be most important for care-recipients. Moreover, it has been demonstrated that care-recipients would like to build a relationship with the individual providing care [4]. Furthermore, a calm nature of the caregiver is important for care-recipients. Please see the ‘Previous research’ section for further details.

This knowledge is important for caregivers as well as nursing services. Knowing the factors which are important for care-recipients might help to reduce the gap between care-recipients’ expectations and reality in professional caregiving at home or nursing home facilities. Our study focused on individuals aged 65 and above as these individuals are at risk of needing long-term care in the near future. Moreover, it was demonstrated that these individuals are well-informed about different aspects of long-term care [13].

## Methods

### Sample

Trained staff interviewed 1006 individuals aged 65 years and over living in private households with conventional telephone connection by phone (Computer Assisted Telephone Interview, approximate duration 25 min) in 2015 [14, 15]. Fieldwork was conducted by Berlin based USUMA—a market research company. By using the Guidelines for Telephone Surveys from the ADM (Arbeitskreis Deutscher Markt- und Sozialforschungsinstitute e.V.), individuals were randomly selected from the community-dwelling older population. Moreover, computer-generated numbers were used in order to allow for ex-directory households as well. Furthermore, repeat calls were made on different days of the week until an answer was obtained. From the gross sample (n = 2346), 1006 interviews were realized (42.9%). Lack of time/lack of interest (12.1%) and refusal to take part in telephone surveys (26.5%) were main reasons for refusal.

When the respondents agreed to complete the interview, oral informed consent was given. Oral consent is common in survey research in Germany. The ethical guidelines of the International Code of Marketing and Social Research Practise by the International Chamber of Commerce and the European Society for Opinion and Marketing Research were followed. Drawing on expert interviews [4] as well as a systematic review of the literature (which is currently under review), a questionnaire was designed to measure long-term care preferences including characteristics of professional caregivers (Additional file 1). Thereby, items were not taken directly from previously published instruments. Instead, items were developed based on existing items because most of the existing items did not fit the structure of a Likert scale. Therefore, items were reformulated. Moreover, the items were adapted to the target group of old age individuals. Thus, the purpose was to create understandable, short and succinct items. According to recent guidelines [16], Likert scales are easy to answer and produce a high response rate among old age individuals.

In order to improve the questionnaire different pretests were done including evaluation conversations and a pilot study. First, evaluation conversations were carried out with experts, guided interviews and telephone interviews with participants not included in the current study. Subsequently, a pretest was done under real life conditions (n = 31) with the target-population. Furthermore, the trained staff from USUMA received a glossary where the items and the underlying intentions were explained in detail.

### Dependent variables

The preferences for characteristics of professional caregivers were quantified as follows: Regarding the characteristics of professional caregivers irrespective of care provided at home or in a nursing home, it is important to me that … (from 1 = “totally agree” to 4 = “totally disagree”):

- … they have the same gender (short (notation used in Table 1 and Table 2): same gender).

**Table 1:** Bivariate associations between independent variables and preferences for nursing home care

	Same gender		German language		Same cultural background		Orderly appearance		Empathetic, kind		Punctual, reliable		Enough time		Small team	
	Low preferences (n = 638; 64.3%)	High preferences (n = 354; 35.7%)	Low preferences (n = 236; 23.5%)	High preferences (n = 767; 76.5%)	Low preferences (n = 452; 45.8%)	High preferences (n = 534; 54.2%)	Low preferences (n = 36; 3.6%)	High preferences (n = 965; 96.4%)	Low preferences (n = 7; 0.7%)	High preferences (n = 998; 99.3%)	Low preferences (n = 18; 1.8%)	High preferences (n = 986; 98.2%)	Low preferences (n = 84; 8.5%)	High preferences (n = 902; 91.5%)	Low preferences (n = 74; 7.5%)	High preferences (n = 911; 92.5%)
Age: Mean (SD)	75.3 (6.4)*	76.2 (7.0)	75.0 (6.4)^+^	75.9 (6.6)	74.8 (6.4) ***	76.3 (6.7)	77.1 (6.4)^+^	75.6 (6.6)	73.7 (7.2)	75.7 (6.6)	75.9 (6.0)	75.7 (6.6)	77.0 (6.6)*	75.5 (6.6)	76.8 (6.7)	75.5 (6.6)
Sex																
Women	263***	299	121**	314	256	302	17	550	3	566	12	556	42	521	35^+^	524
Men	375	55	115	453	196	232	19	415	4	432	6	430	42	381	39	387
Living situation																
Living with partner or spouse	280***	106	94	297	165	220	11	380	1	391	5	387	25^+^	360	41	556
Others	358	248	142	470	287	314	25	585	6	607	13	599	59	542	33	355
Region																
West Germany	484	256	183	565	367***	370	26	720	7	742	17^+^	731	67	669	57	677
East Germany	154	98	53	202	85	164	10	245	0	256	1	255	17	233	17	234
Education																
Without a vocational degree	33***	39	12***	62	36*	36	4	71	1	74	2	73	10*	63	7**	64
Apprenticeship, full-time vocational school	202	170	67	313	148	223	15	362	3	377	5	374	20	350	17	355
Professional school or trade and technical school for vocational education	156	86	58	185	110	129	5	238	2	241	8	235	24	216	16	224
University, school of engineering	242	58	98	202	156	142	12	288	1	300	3	298	30	267	34	262
Place of birth																
Born in Germany	594	322	216	709	410	498	33	890	7	920	17	909	79	830	67	842
Born abroad	42	31	20	55	41	34	3	72	0	75	1	74	5	69	7	66
Having children																
Yes	526	301	187*	650	364*	459	31	805	5	834	15	823	64^+^	760	57	764
No	111	53	49	116	87	75	5	159	2	163	3	162	20	141	17	146
Status of health insurance																
Statutory health insurance	518***	326	188**	666	377	463	32	821	7	849	18^+^	837	68	773	58^+^	781
Private health insurance	116	28	47	98	73	69	4	140	0	145	0	145	16	125	16	126
Provided care for family/friends																
Yes	320^+^	157	108	373	251*	259	14	504	2	519	11	510	35	437	42	470
No	317	197	128	393	200	275	22	460	5	478	7	475	49	464	32	440
Level of care																
Yes	29**	31	10	50	21	37	3	57	0	60	0	60	5	55	6	53
No	607	322	224	716	429	496	31	907	6	936	17	924	78	845	67	856
Self-rated health (from ‘very bad’ to ‘very good’): Mean (SD)	3.7 (0.9)*	3.5 (0.9)	3.8 (0.9)**	3.6 (0.9)	3.7 (0.9)**	3.5 (0.9)	3.4 (1.2)	3.6 (0.9)	4.0 (1.4)	3.6 (0.9)	3.7 (1.0)	3.6 (0.9)	3.6 (1.0)	3.6 (0.9)	3.6 (0.9)	3.6 (0.9)
Involvement in the issue of need for care, from ‘very little’ to ‘very much’): Mean (SD)	2.9 (1.4)	3.0 (1.4)	2.8 (1.5)	2.9 (1.4)	3.0 (1.5)*	2.8 (1.4)	3.1 (1.6)	2.9 (1.4)	3.1 (1.7)	2.9 (1.4)	3.0 (1.7)	2.9 (1.4)	2.6 (1.4)*	2.9 (1.4)	2.8 (1.6)	2.9 (1.4)

Comparisons between the two groups were done using *t* test and Chi square procedures. ^+^.10, *.05, **.01, ***.001

**Table 2:** Predictors of preferences for nursing home care. Results of logistic regressions (for each outcome measure: 0 = low preferences; 1 = high preferences)

Independent variables	(1)	(2)	(3)	(4)	(5)	(6)	(7)	(8)
	Same gender	German language	Same cultural background	Orderly appearance	Empathetic, kind	Punctual, reliable	Enough time	Small team
Age	1.000	1.006	1.032**	0.972	1.115	1.007	0.969	0.976
	(0.977–1.023)	(0.982–1.030)	(1.011–1.054)	(0.921–1.027)	(0.955–1.301)	(0.935–1.084)	(0.933–1.007)	(0.939–1.013)
Sex (Ref.: male)	8.270***	1.511*	0.985	2.193^+^	5.374^+^	1.232	1.931*	1.547
	(5.640–12.13)	(1.062–2.150)	(0.724–1.340)	(0.921–5.222)	(0.810–35.63)	(0.399–3.801)	(1.068–3.491)	(0.870–2.751)
Living situation (Ref.: Living with partner or spouse)	0.716*	0.753	0.712*	0.524	0.276	0.615	0.527*	0.920
	(0.514–0.997)	(0.534–1.062)	(0.530–0.955)	(0.230–1.192)	(0.0334–2.281)	(0.202–1.874)	(0.296–0.941)	(0.529–1.600)
West and East Germany (Ref.: East Germany)	0.957	0.896	0.524***	1.236		0.209	0.899	0.791
	(0.648–1.412)	(0.591–1.358)	(0.364–0.754)	(0.491–3.114)		(0.0277–1.574)	(0.450–1.797)	(0.394–1.587)
Apprenticeship, full-time vocational school (Ref.: Without a vocational degree)	1.188	0.955	1.304	1.583	4.939	2.332	3.141*	2.094
	(0.683–2.066)	(0.478–1.909)	(0.767–2.216)	(0.492–5.096)	(0.406–60.12)	(0.414–13.14)	(1.306–7.556)	(0.809–5.422)
Professional school or trade and technical school for vocational education	0.862	0.714	0.979	3.893^+^	5.611	0.881	1.832	1.660
	(0.477–1.556)	(0.350–1.457)	(0.558–1.715)	(0.830–18.26)	(0.353–89.29)	(0.167–4.635)	(0.757–4.435)	(0.614–4.486)
University, Fachhochschule, school of engineering	0.609	0.514^+^	0.750	1.319	17.87	1.301	2.101	0.810
	(0.327–1.135)	(0.251–1.053)	(0.423–1.331)	(0.349–4.993)	(0.476–670.3)	(0.202–8.378)	(0.831–5.312)	(0.313–2.094)
German-born (Ref.: no)	0.555*	1.115	1.481	1.325		0.396	0.497	1.987^+^
	(0.323–0.953)	(0.643–1.933)	(0.918–2.388)	(0.423–4.153)		(0.0429–3.657)	(0.165–1.501)	(0.963–4.099)
Children (Ref.: No children)	1.024	1.208	1.183	0.854	1.911	0.868	1.610	1.432
	(0.672–1.559)	(0.800–1.824)	(0.818–1.709)	(0.303–2.403)	(0.260–14.06)	(0.232–3.247)	(0.869–2.982)	(0.765–2.681)
Status of health insurance (Ref.: statutory health insurance)	0.625^+^	0.772	0.938	1.162			0.738	0.874
	(0.382–1.023)	(0.513–1.161)	(0.641–1.372)	(0.372–3.625)			(0.371–1.471)	(0.462–1.655)
Provided care for family/friends (Ref.: no)	0.840	0.737^+^	0.845	1.637	1.843	0.390^+^	0.520*	0.670
	(0.609–1.160)	(0.531–1.024)	(0.638–1.120)	(0.754–3.552)	(0.274–12.41)	(0.131–1.161)	(0.304–0.891)	(0.398–1.128)
Level of care (Ref.: no)	0.622	1.026	0.787	0.734			1.005	1.737
	(0.328–1.182)	(0.494–2.132)	(0.430–1.437)	(0.182–2.954)			(0.321–3.146)	(0.645–4.677)
Self-rated health (from ‘very bad’ to ‘very good’)	0.882	0.877	0.863^+^	1.409^+^	0.696	1.050	1.089	1.009
	(0.740–1.053)	(0.734–1.047)	(0.741–1.005)	(0.953–2.084)	(0.264–1.832)	(0.591–1.868)	(0.816–1.454)	(0.762–1.337)
Involvement in the issue need for care (from ‘very little’ to ‘very much’)	0.953	1.064	0.908^+^	0.827	1.065	1.110	1.344**	1.167^+^
	(0.851–1.066)	(0.949–1.192)	(0.823–1.003)	(0.634–1.079)	(0.558–2.033)	(0.780–1.581)	(1.109–1.630)	(0.971–1.401)
Constant	0.350	3.240	0.544	32.37	0.00193	204.3	31.01	5.131
	(0.0231–5.299)	(0.188–55.86)	(0.0481–6.146)	(0.0809–12,946)	(3.40e−09–1095)	(0.142–294,745)	(0.358–2685)	(0.0745–353.6)
Observations	974	983	967	982	521	788	967	967
Pseudo R^2^	0.178	0.036	0.046	0.067	0.145	0.061	0.073	0.056

Odd ratios were reported. 95% confidence intervals in parentheses

Region was dropped (253 observations not used) since it predicts success perfectly. Place of birth was dropped (58 observations not used) since it predicts success perfectly. Status of health insurance was dropped (121 observations not used) since it predicts success perfectly. Level of care was dropped (32 observations not used) since it predicts success perfectly. Status of health insurance was dropped (141 observations not used) since it predicts success perfectly. Level of care was dropped (55 observations not used) since it predicts success perfectly. Observations with missing values were dropped (listwise deletion). *** p < 0.001, ** p < 0.01, * p < 0.05, ^+^ p < 0.10
- … they have very good German language skills (short: German language).
- … they share the same cultural background (short: same cultural background).
- … they show an orderly appearance (short: orderly appearance).
- … they are empathetic, kind, understanding (short: empathetic, kind).
- … they are punctual and reliable (short: punctual, reliable).
- … they have enough time (going beyond physical care, e.g., for conversation) (short: enough time).
- … the nursing team is small (short: small team).

The dependent variables were dichotomized (0 “totally disagree” and “rather disagree”; 1 “totally agree” and “rather agree”) to reflect high preferences versus low preferences. For the sake of readability, the terms “importance” and “preferences” have been used interchangeably in our study because we assume that these factors are highly correlated.

### Independent variables

Socioeconomic variables were used as follows: age, sex, whether they have children or not (Ref.: no children), whether they were born in Germany or not (Ref.: not born in Germany), West and East Germany (Ref.: East Germany), living situation (Ref.: living with partner or spouse; others (living alone; living with other family members; living with other individuals)), status of health insurance (Ref.: statutory health insurance; private health insurance), and education (Ref.: without a vocational degree; others (apprenticeship, full-time vocational school; professional school or trade and technical school for vocational education; University, school of engineering)).

Moreover, individuals were asked whether they have ever provided informal care for family or friends (Ref.: no). As for health status, self-rated health (from 1 “very bad” to 5 “very good”) and level of care (Ref.: no) were included as independent variables. Recipients are classified into three levels of care (depending on the care required) based on an assessment by a nurse or a physician of the medical service of the German statutory health insurance system. The need of care was dichotomized (with 0 no level of care; 1 level 1 to 3). Furthermore, the involvement in the issue of need for care (“How much have you thought about the issue of ‘need for care’”) was assessed, ranging from 1 (“very little”) to 5 (“very much”).

### Statistical analysis

Bivariate associations between preferences (high preferences; low preferences) and independent variables were analyzed using t-tests, Chi square and Fisher’s exact tests, as appropriate. Logistic regressions were used to investigate the relationship between our numerous independent variables and the preference outcomes. Eight logistic regressions models were computed. Outcome variables were (preferences for…): (i) Same gender, (ii) German language, (iii) Same cultural background, (iv) Orderly appearance, (v) Empathetic, kind, (vi) Punctual, reliable, (vii) Enough time, (viii) Small team. In each model, independent variables were age, sex, living situation, region, education, place of birth, having children, status of health insurance, provided care for family/friends, level of care, self-rated health, and involvement in the issue of need for care.

In additional analysis, we used penalized maximum likelihood logistic regression [17, 18]. It can be used when some of the cells formed by the outcome and categorical predictor variable have no observations [19, 20]. The statistical significance was defined as p value of 0.05 or smaller. All statistical analyses were performed using Stata 14.0 (StataCorp, College Station, Texas). Given that our sample size was sufficiently large (n = 1006 individuals), at least medium effects can be detected.

## Results

### Bivariate associations

**Fig. 1:**
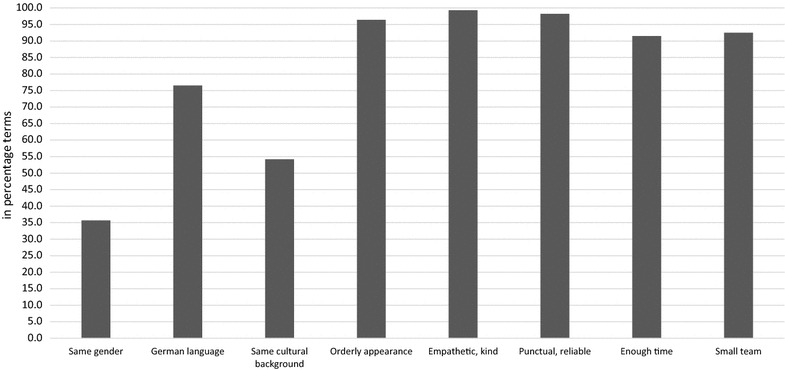
Preferences for nursing home care

Table 1 displays sample characteristics by preferences (low preferences vs. high preferences). The preferences were highest for ‘empathetic, kind’ (99.3%), ‘punctual, reliable’ (98.2%), ‘orderly appearance’ (96.4%), ‘small team’ (92.5%) and ‘enough time’ (91.5%) (Please see also Fig. 1). Moreover, most of the individuals (76.5%) reported high preferences for ‘German language’, whereas the preferences were markedly lower for ‘same cultural background’ (54.2%) and ‘same gender’ (35.7%).

Preferences for ‘same gender’ were significantly associated with age, gender, living situation, education, status of health insurance, need of care, and self-rated health. Preferences for ‘German language’ were significantly associated with gender, education, having children, status of health insurance, and self-rated health. Preferences for ‘same cultural background’ were significantly associated with age, region, having children, provided care for family/friends, self-rated health, and involvement in the issue of need for care. Neither preferences for ‘orderly appearance’, nor preferences for ‘empathetic, kind’, nor preferences for ‘punctual, reliable’ were significantly associated with included predictors. Preferences for ‘enough time’ were significantly associated with age, education, and involvement in the issue of need for care. Preferences for ‘small team’ were significantly associated with education.

### Regression analysis

Results of multiple logistic regressions are depicted in Table 2.

Preferences for ‘same gender’ were positively associated with being female [OR 8.3 (5.6–12.1)], living with partner or spouse [OR 1.4 (1.0–1.9)], and being born abroad [OR 1.8 (1.1–3.1)]. Preferences for ‘German language’ were positively associated with being female [OR 1.5 (1.1–2.1)]. Preferences for ‘same cultural background’ were positively associated with age [OR 1.0 (1.0–1.1)], living with partner or spouse [OR 1.4 (1.0–1.9)], and East Germany [OR 1.9 (1.3–2.7)]. While preferences for ‘orderly appearance’, ‘empathetic, kind’, ‘punctual, reliable’ as well as ‘small nursing team’ were all not significantly associated with included independent variables, preferences for ‘enough time’ were positively associated with being female [OR 1.9 (1.1–3.5)], living with partner or spouse [OR 1.9 (1.1–3.4)], education [Apprenticeship, full-time vocational school, OR 3.1 (1.3–7.6)], not providing care for family/friends [OR 1.9 (1.1–3.3)], and involvement in the issue of need for care [OR 1.3 (1.1–1.6)].

Since some of the cells formed by the outcome and categorical predictor variable have no observations, we repeated our estimates with penalized maximum likelihood logistic regressions (instead of logistic regressions) in additional analysis. In terms of effect sizes and significance, our findings were comparable to those from the main logistic regressions (results not shown but available upon request).

## Discussion

### Main findings

By using a large, population-based survey in individuals aged 65 and above in Germany, we aimed at investigating which factors are associated with characteristics of professional long-term caregivers among individuals in old age. More specifically, the predictors of preferences for characteristics of caregivers were examined.

The preferences were highest for ‘empathetic, kind’ (99.3%), ‘punctual, reliable’ (98.2%), ‘orderly appearance’ (96.4%), ‘small team’ (92.5%) and ‘enough time’ (91.5%). Moreover, most of the individuals (76.5%) reported high preferences for ‘German language’, whereas the preferences were markedly lower for ‘same cultural background’ (54.2%) and ‘same gender’ (35.7%).

In multiple logistic regressions, preferences for ‘same gender’ were positively associated with being female, living with partner or spouse, and being born abroad. Preferences for ‘German language’ were positively associated with being female. Preferences for ‘same cultural background’ were positively associated with age, living with partner or spouse, and East Germany. Preferences for ‘orderly appearance’, ‘empathetic, kind’, ‘punctual, reliable’ and ‘small nursing team’ were all not significantly associated with included independent variables, whereas preferences for ‘enough time’ were positively associated with being female, living with partner or spouse, education, not providing care for family/friends, and involvement in the issue of need for care.

### Previous research

As already found in the literature [9, 10], soft skills of caregivers such as empathy, kindness, punctuality or reliability are most important for care-recipients. This is also in line with a recent study [4] showing that individuals in need for care wish to build a relationship with the caregiver. Moreover, the caregiver should do their tasks and activities with calm and in a leisurely way [4]. Besides, the high preferences for a small nursing team might be explained by the perception of individuals that small teams are associated with deep personal relationships as well as soft skills such as trust or feelings of emotional attachment which are highly important for individuals in need for care [4]. Compared with soft skills, we found that other factors such as ‘same cultural background’ and ‘same gender’ are far less important. This might be mainly explained by the fact that these preferences, unlike soft skills, do not reflect basic caregiving needs. This corresponds to the findings of van Haitsma and colleagues [21].

Being female was positively associated with preferences for ‘same gender’ and preferences for ‘German language’. This is also in line with previous studies reporting that women prefer a general practitioner of the same gender [22–25]. Moreover, this is also in accordance with a previous qualitative study among 60 older lesbian, gay, bisexual and queer (LGB) individuals in which Westwood [26] found that older LGB women might be more likely to prefer gender-specific care. Our findings might be explained by the fact that women experience increased levels of stress when getting intimate care by men [27]. In addition, women might fear that quality of care and conversations suffer when caregivers have poor skills in the German language. This might explain why preferences for ‘German language’ were positively associated with being female.

Living with partner was positively associated with preferences for ‘same gender’ and preferences for ‘same cultural background’. Consequently, individuals living with partner might be more afraid of care provided by the opposite gender. This might be explained by the fact that individuals in old age living with partner could not imagine that other individuals apart from their wife or husband provide assistance with basic activities of daily living such as toileting or bathing.

In addition, preferences for ‘same cultural background’ were positively associated with age and East Germany. These associations might reflect differences in cultural values [28–30]. Different preferences for ‘same racial/ethnic group’ regarding health care providers were also reported among different ethnic groups (Asian-Americans compared to non-Latino Whites) [31].

The non-significant associations between ‘orderly appearance’, ‘empathetic, kind’, ‘punctual, reliable’ as well as ‘small nursing team’ and included predictors might be mainly explained by the fact that nearly every individual wishes to have them in the future. Thus, these preferences for characteristics of professional caregivers might be generally seen as basic (human) needs.

The positive association between preferences for ‘enough time’ (going beyond physical care, e.g., for conversation) and being female, higher education as well as living with partner or spouse might be explained by the greater need for social interactions in these groups [32, 33].

Furthermore, while the positive association between involvement in the issue of need for care and preferences for ‘enough time’ might be explained by the fact that a high involvement in this issue is associated with a higher preference for soft skills of caregivers, the positive association between not providing care and the preferences for ‘enough time’ was quite unexpected and might be explained by unobserved factors associated with providing informal care (for example, personality traits [34–36]). In addition, this association might be explained by the fact that individuals who already provided informal care have a more realistic perspective on life in a long-term care setting. Consequently, these individuals might be more aware of the organizational and time constraints faced by caregiver and that “enough time” (not closely related to caregiving aspects, e.g. time for conversations) will likely place additional burden on caregivers. However, further research is required to clarify this relationship.

### Strengths and limitations

It should be highlighted that our data were derived from a large, population-based sample among individuals in old age. Moreover, numerous important independent and dependent variables were captured. For example, adjusting for numerous potential confounders, we provide novel evidence that region (West and East Germany) is differentially associated with preferences for ‘same cultural background’. However, our study also has some limitations. This is a cross-sectional study. Therefore, temporal relationships cannot be determined. Future studies aimed at examining the long-term impact of predictors on long-term care preferences are needed. In addition, upcoming studies should validate the instruments used in our study. In addition, the self-reported data might suffer some degree of inaccuracy.

## Conclusions

Our data suggest that it is important to almost every individual aged 65 and above in Germany that professional long-term caregivers are (i) empathetic, kind, and understanding, (ii) punctual and reliable, (iii) have enough time (e.g., for conversation), (iv) and have an orderly appearance. Furthermore, high preferences for skills in German language were reported. Moreover, it is important to them to be cared for in a small team. Characteristics such as having the same cultural background or having the same gender are less important. Furthermore, several factors such as gender or region were found to be associated with characteristics of caregivers in nursing home facilities. By knowing these factors, nursing services can tailor their activities to the needs of care-recipients. Reducing the gap between caregivers’ needs and reality might in turn help to increase the satisfaction of care-recipients in nursing home facilities.

## Additional file

**Additional file 1.** Questionnaire.

## Abbreviations

ADM: Arbeitskreis Deutscher Markt- und Sozialforschungsinstitute
CATI: Computer Assisted Telephone Interview
OR: odds ratio
USUMA: Unabhängige Serviceeinrichtung für Umfragen, Methoden und Analysen
