# Supplementary material for: Who should take care of me? Preferences of old age individuals for characteristics of professional long-term caregivers: an observational cross-sectional study
Source: BMC Res Notes. 2017 Aug 10;10:382. doi: 10.1186/s13104-017-2717-3 (PMC5553919; doi:10.1186/s13104-017-2717-3)
Supplement: Supplementary file 1 — Additional file 1. Questionnaire. [file 13104_2017_2717_MOESM1_ESM.docx]

| **Bevor wir zu Fragen zu Ihren Wünschen kommen, benötige ich ein paar Angaben zu Ihrer Person:** | | | | | | | | |
| --- | --- | --- | --- | --- | --- | --- | --- | --- |
|  | **Wie alt sind Sie?** | ___________ **Jahre** | | | | | | |
|  | **Welches Geschlecht haben Sie?** | **Weiblich** | | **Männlich** | | | | |
|  | **Wie sehr haben Sie sich bisher mit dem Thema Pflegebedürftigkeit auseinandergesetzt?** | **Sehr viel** | **Viel** | | **Mittel-mäßig** | **Wenig** | **Sehr wenig** | **Weiß nicht** |
|  | **Haben Sie zurzeit eine Pflegestufe, sind also pflegebedürftig im Sinne der sozialen Pflegeversicherung?** | **Nein**  **Ja** | | | | | | |
|  | **Falls ja, welche Pflegestufe ist dies und durch wen werden Sie versorgt bzw. gepflegt?** (Weiter mit Frage 6)  **Pflegestufe: 0 1** **2** **3**  **Versorgende Person/en:**  **______________________________________________________________________________________** | | | | | | | |
|  | **Wie hoch schätzen Sie Ihr persönliches Risiko ein, einmal pflegebedürftig zu werden?** | **Sehr groß** | **Eher groß** | | **Mittel** | **Eher gering** | **Sehr gering** | **Weiß nicht** |
|  | **Wie schätzen Sie Ihren Gesundheitszustand im Allgemeinen ein?** | **Sehr gut** | **Eher gut** | | **Mittel-mäßig** | **Eher schlecht** | **Sehr schlecht** | **Weiß nicht** |
|  | **Haben Sie bereits konkrete Maßnahmen für den Fall einer Pflegebedürftigkeit geplant bzw. falls Ihr Pflegebedarf sich vergrößern sollte?**  (Z.B. Hausumbau, Umzug zu Angehörigen oder in ein Heim etc.) | **Nein**  **Ja** | | | | | | |
|  | **Falls ja, welche Maßnahmen sind dies?** | | | | | | | |
|  | **Haben Sie bereits selbst Angehörige oder Personen aus Ihrem Umfeld gepflegt?** | **Nein**  **Ja** | | | | | | |
|  | **Falls ja, wen betraf dies und für wie lange haben Sie gepflegt?**  **Gepflegte Person/en: ____________________________________________________________________**  **Dauer (Monate/Jahre): _______________________________________________________________** | | | | | | | |

| **Im Folgenden geht es um Ihre konkreten Wünsche bei Pflegebedürftigkeit. Bitte geben Sie an, inwiefern die Aussagen auf Ihre Vorstellungen zutreffen, auch wenn Sie bereits eine bestimmte Form der Versorgung in Anspruch nehmen sollten. Bitte antworten Sie immer mit:**  ***„Trifft voll und ganz zu“* oder *„Trifft eher zu“* bzw. *„Trifft eher nicht zu“* oder *„Trifft überhaupt nicht zu“.*** | | | | | | | | | |
| --- | --- | --- | --- | --- | --- | --- | --- | --- | --- |
| Bitte angeben, falls Wohnform nicht bekannt ist!  **Ort der Versorgung:**  **Wenn ich Unterstützung benötigen sollte,**  **möchte ich nach Möglichkeit …** | | **Trifft voll und ganz zu** | **Trifft eher zu** | | **Trifft eher nicht zu** | | **Trifft überhaupt nicht zu** | | **Nicht bekannt** |
|  | **… in meinen eigenen vier Wänden gepflegt werden.** |  |  | |  | |  | | - |
|  | **… im Haus oder in der Wohnung meiner Angehörigen gepflegt werden.** |  |  | |  | |  | | - |
|  | **… im Betreuten Wohnen bzw. Servicewohnen gepflegt werden.** |  |  | |  | |  | |  |
|  | **… in einem Alten- oder Pflegeheim gepflegt werden.** |  |  | |  | |  | |  |
|  | **… im Ausland gepflegt werden.** |  |  | |  | |  | |  |
| **Nun geht es nur um die Häusliche Pflege und welche Versorgung Sie dort bevorzugen würden, falls Sie auf Pflege angewiesen sein sollten. Inwieweit treffen die folgenden Aussagen für Sie zu?** | | | | | | | | | |
| **Art der Häuslichen Pflege:**  **Wenn ich bei mir oder Angehörigen zu Hause auf Pflege angewiesen sein sollte, möchte ich nach Möglichkeit …** | | **Trifft voll und ganz zu** | | **Trifft eher zu** | | **Trifft eher nicht zu** | | **Trifft überhaupt nicht zu** | |
|  | **… ausschließlich von Personen aus meinem Umfeld gepflegt werden (z.B. Partner, Kinder, Freunde, Bekannte).** |  | |  | |  | |  | |
|  | **… ausschließlich von einem professionellen Pflegedienst versorgt werden.** |  | |  | |  | |  | |
|  | **… eine Mischung aus professioneller Pflege (also einem Pflegedienst) und Pflege durch Familie, Freunde etc. erhalten.** |  | |  | |  | |  | |
|  | **… eine „Rund-um-die-Uhr-Betreuung“ erhalten, z.B. durch eine privat bezahlte Pflegeperson.** |  | |  | |  | |  | |
| **Zusätzliche Dienste und Angebote:**  **Wenn ich bei mir oder bei Angehörigen zu Hause versorgt werde, wäre es mir wichtig, …** | | **Trifft voll und ganz zu** | | **Trifft eher zu** | | **Trifft eher nicht zu** | | **Trifft überhaupt nicht zu** | |
|  | **… dass meine Mahlzeiten ins Haus geliefert werden, z.B. „Essen auf Rädern“.** |  | |  | |  | |  | |
|  | **… hauswirtschaftliche Hilfen in Anspruch zu nehmen, z.B. für Reinigung, Einkauf etc.** |  | |  | |  | |  | |
|  | **… eine Betreuung oder Alltagsbegleitung zu haben, z.B. zur Beschäftigung, Unterhaltung etc.** |  | |  | |  | |  | |
|  | **… einen Fahrdienst nutzen zu können.** |  | |  | |  | |  | |
|  | **… einen Hausnotruf bzw. ein Notrufsystem zu haben.** |  | |  | |  | |  | |
| **Nun geht es nur um die Pflege in einer stationären Einrichtung, falls Sie einmal auf Pflege in einem Alten- oder Pflegeheim angewiesen sein sollten. Inwieweit treffen die folgenden Aussagen für Sie zu?** | | | | | | | | | |
| **Art der Unterbringung:**  **Mir ist wichtig, …** | | **Trifft voll und ganz zu** | | **Trifft eher zu** | | **Trifft eher nicht zu** | | **Trifft überhaupt nicht zu** | |
|  | **… in einem eigenen Zimmer zu wohnen, also es nicht mit anderen Bewohnern zu teilen.** |  | |  | |  | |  | |
|  | **… dass ich ein Haustier halten kann bzw. Haustiere erlaubt sind.** |  | |  | |  | |  | |
|  | **… dass ich rund um die Uhr Besuch empfangen kann.** |  | |  | |  | |  | |
|  | **… in einem kleinen Pflegeheim zu wohnen, also bis max. 50 Bewohner.** |  | |  | |  | |  | |
| **Lage des Pflegeheims:**  **Wenn ich in einem Pflegeheim versorgt werden sollte, möchte ich …** | | **Trifft voll und ganz zu** | | **Trifft eher zu** | | **Trifft eher nicht zu** | | **Trifft überhaupt nicht zu** | |
|  | **… dass das Pflegeheim so nah wie möglich an meinem jetzigen Wohnort liegt.** |  | |  | |  | |  | |
|  | **… dass das Pflegeheim so nah wie möglich an dem Wohnort meiner nächsten Angehörigen liegt.** |  | |  | |  | |  | |
|  | **… dass es in einer Stadt liegt.** |  | |  | |  | |  | |
| **Aktivitäten und Tagesgestaltung:**  **In einem Pflegeheim ist mir wichtig, …** | | **Trifft voll und ganz zu** | | **Trifft eher zu** | | **Trifft eher nicht zu** | | **Trifft überhaupt nicht zu** | |
|  | **… regelmäßig an *körperlichen* Aktivitäten teilzunehmen, z.B. Bewegungsangebote, Tanz- und Fitnesskurse, Gartenarbeit o.ä.** |  | |  | |  | |  | |
|  | **… regelmäßig an *künstlerisch-gestalterischen* Aktivitäten teilzunehmen, z.B. Handwerken, Malen, Basteln, Handarbeit o.ä.** |  | |  | |  | |  | |
|  | **… regelmäßig an *sozialen* Aktivitäten teilzunehmen, z.B. Lesezirkel, Koch- und Backkurse, Begegnungen (mit Kindergärten, Schulklassen, Behindertenwerkstätten) o.ä.** |  | |  | |  | |  | |
|  | **… regelmäßig an Ausflügen teilzunehmen, z.B. Besuche im Museum, Theater, Kino, Zoo o.ä.** |  | |  | |  | |  | |
| **Im Folgenden geht es nur um die Eigenschaften von *professionellen* Pflegekräften und um die Qualität der Versorgung, egal ob bei der Pflege zu Hause oder in einem Pflegeheim.** | | | | | | | | | |
| **Eigenschaften der Pflegekräfte:**  **Bei den Pflegekräften, die mich versorgen, ist mir wichtig, …** | | **Trifft voll und ganz zu** | | **Trifft eher zu** | | **Trifft eher nicht zu** | | **Trifft überhaupt nicht zu** | |
|  | **… dass sie das gleiche Geschlecht haben wie ich.** |  | |  | |  | |  | |
|  | **… dass sie sehr gute Deutschkenntnisse besitzen.** |  | |  | |  | |  | |
|  | **… dass sie dem gleichen Kulturkreis angehören wie ich.** |  | |  | |  | |  | |
|  | **… dass sie ein ordentliches äußeres Erscheinungsbild haben.** |  | |  | |  | |  | |
|  | **… dass sie sehr einfühlsam, freundlich und verständnisvoll sind.** |  | |  | |  | |  | |
|  | **… dass sie sehr pünktlich und zuverlässig sind.** |  | |  | |  | |  | |
|  | **… dass sie genügend Zeit über meine kör-perliche Versorgung hinaus haben, z.B. für Gespräche oder kleine HilfenHilfen im Haushalt.** |  | |  | |  | |  | |
|  | **… dass das Pflegeteam klein und überschaubar ist.** |  | |  | |  | |  | |
| **Qualität der Pflege:**  **Bei der Versorgung und Pflege ist mir wichtig, …** | | **Trifft voll und ganz zu** | | **Trifft eher zu** | | **Trifft eher nicht zu** | | **Trifft überhaupt nicht zu** | |
|  | **… dass ich mich gut versorgt fühle, unabhängig von der Qualifikation der Pflegekräfte.** |  | |  | |  | |  | |
|  | **… dass die Pflegekräfte fachlich hoch qualifiziert sind.** |  | |  | |  | |  | |
| **Nun folgen noch einige allgemeine Angaben zu Ihren Wünschen.** | | | | | | | | | |
| **Ihre persönliche Unabhängigkeit betreffend:**  **Mir ist es im Allgemeinen sehr wichtig, …** | | **Trifft voll und ganz zu** | | **Trifft eher zu** | | **Trifft eher nicht zu** | | **Trifft überhaupt nicht zu** | |
|  | **… dass ich wichtige pflegebezogene Entscheidungen immer in Absprache mit vertrauten Personen treffe, z.B. Angehörige, Hausarzt etc.** |  | |  | |  | |  | |
|  | **… selbst festzulegen, welche Gerichte und Lebensmittel es zu essen gibt.** |  | |  | |  | |  | |
|  | **… wann ich ins Bett gehe und wie lange ich schlafen kann.** |  | |  | |  | |  | |
|  | **… dass meine Wohnung bzw. mein Zimmer sehr ordentlich und gepflegt ist.** |  | |  | |  | |  | |
|  | **… dass ich meinen Wohnraum individuell gestalten kann, z.B. durch eigene Möbel und Gegenstände.** |  | |  | |  | |  | |

| **Zum Ende des Fragebogens folgen abschließend noch einmal einige Fragen zu Ihrer Person.** | | | | |
| --- | --- | --- | --- | --- |
| 1. **Wie ist Ihre aktuelle Wohnsituation? Leben Sie … ?** | | **… allein**  **… mit festem Partner oder Ehepartner**  **… mit anderen Familienmitgliedern**  **… mit anderen Personen** | | |
| 1. **Wohnen Sie … ?** | | **… in Ihrem Eigenheim oder Eigentumswohnung**  **… zur Miete**  **… bei Angehörigen** | | |
| 1. **Wie viele Personen leben in Ihrem Haushalt, Sie eingeschlossen?** | | **_______ Personen; davon jünger als 15 Jahre: ______ Personen** | | |
| 1. **Welche Postleitzahl hat Ihr Wohnort?** | | **PLZ: _________________** | | |
| 1. **Was ist Ihr höchster berufsbildender Abschluss?** | | **Ohne berufsbildenden Abschluss**  **Berufsschule/Lehre**  **Fachschule/Technikerschule/Meisterschule**  **Universität/Fachhochschule/Ingenieurschule/Polytechnikum**  **Anderer Abschluss, und zwar: ­­­­­­­­­­­­­­­­­­­­­­­­­­­­­­­___________________________** | | |
| 1. **Sind Sie verrentet?** | | **Nein**  **Ja** | | |
| 1. **Wie sind Sie krankenversichert?** | | **Gesetzliche Krankenversicherung**  **Private Krankenversicherung** | | |
| 1. **Haben Sie eine Pflegezusatzversicherung abgeschlossen oder anderweitig finanziell für eine Pflegebedürftigkeit vorgesorgt?** | | **Nein**  **Ja** | | |
| 1. **Haben Sie das Gefühl, dass Sie für eine Pflegebedürftigkeit ausreichend vorgesorgt haben?** | | **Nein**  **Ja** | | |
| 1. **Haben Sie Kinder?** | | **Nein**  **Ja Wie viele? _____ Tochter/Töchter; _____ Sohn/Söhne** | | |
| **Falls ja, wie häufig haben Sie schätzungsweise im Monat Kontakt zu Ihren Kindern, z.B. telefonisch oder Besuche?** | | | **______________ x/Monat** | |
| 1. **Haben Sie andere Verwandte und Freunde, zu denen ein enger Kontakt besteht?** | | **Nein**  **Ja** | | |
| **Falls ja, wie häufig haben Sie Kontakt zu diesen Personen, z.B. telefonisch oder Besuche?** | | | **______________ x/Monat** | |
| 1. **Gibt es Personen, die sich für den Fall, dass Sie pflegebedürftig werden sollten, um Sie kümmern können?** | | **Nein**  **Ja** | | |
| **Falls ja, wer wäre dies?** (Angabe mehrerer Personen möglich) | | | | |
|  | **Wurden Sie in Deutschland geboren?** | **Nein**  **Ja** | | |
|  | **Falls nein, aus welchem Land stammen Sie und wie lange leben Sie bereits in Deutschland?**  **Herkunftsland: _______________________________________________________________**  **In Deutschland seit: _______________ Jahren** | | | |
| 1. **Wie hoch ist in etwa das monatliche Netto-Einkommen Ihres Haushalts insgesamt?**   **Bitte zählen Sie das Netto-Einkommen (Einkommen abzüglich Steuern und Sozialversicherungsbeiträgen) sämtlicher Einkommensquellen für alle Haushaltsmitglieder zusammen.** | | **Unter € 500**  **€ 500 bis unter € 750**  **€ 750 bis unter € 1000**  **€ 1000 bis unter € 1500** | | **€ 1500 bis unter € 2000**  **€ 2000 bis unter € 3000**  **€ 3000 bis unter € 5000**  **€ 5000 und mehr** |
